# Real-world experience in initiation of treatment with the selective cardiomyosin inhibitor mavacamten in an outpatient clinic cohort during the 12-week titration period

**DOI:** 10.1007/s00392-024-02544-w

**Published:** 2024-10-08

**Authors:** Finn Becker, Julia Novotny, Nadine Jansen, Sebastian Clauß, Florian Möller-Dyrna, Birge Specht, Madeleine Orban, Steffen Massberg, Stefan Kääb, Daniel Reichart

**Affiliations:** 1 Department of Medicine I, LMU University Hospital, LMU Munich, Marchioninistrasse 15, 81377 Munich, Germany; 2 DZHK (German Center for Cardiovascular Research), Partner Site Munich, Munich Heart Alliance (MHA), 80802 Munich, Germany; 3 Member of the European Reference Network for Rare, Low Prevalence and Complex Diseases of the Heart (ERN GUARD-Heart), Munich, Germany; 4 Institute of Surgical Research at the Walter-Brendel-Centre of Experimental Medicine University Hospital, LMU Munich, Munich, Germany; 5 Interfaculty Center for Endocrine and Cardiovascular Disease Network Modelling and Clinical Transfer (ICONLMU), LMU Munich, Munich, Germany

**Keywords:** Obstructive hypertrophic cardiomyopathy (oHCM), Treatment, Cardiomyosin inhibitor, Mavacamten, Titration period

## Abstract

**Introduction:**

Lately, mavacamten emerged as a new therapeutic option for symptomatic patients with obstructive hypertrophic cardiomyopathy (oHCM). Clinical trials revealed reduction of serum biomarkers, and left ventricular outflow tract (LVOT) obstruction, as well as an improvement in clinical symptoms and exercise capacity. Nevertheless, clinical experience and manageability of patients in a real-world setting is still lacking.

**Material and methods:**

22 patients with symptomatic oHCM (54.5% male, age 58.5 ± 16.2 years) and elevated LVOT gradients were started on mavacamten between March 2023 and June 2024. All patients were New York Heart Association (NYHA) class II or higher. Seven patients were excluded from primary analysis due to comedication with Angiotensin-converting-enzyme-inhibitors or Angiotensin-II receptor blockers. Cardiac imaging, laboratory work-up and clinical evaluation were assessed at three visits during the 12 weeks initiation phase; Dosing of mavacamten was adjusted according to manufacturer’s recommendations.

**Results:**

At 12 weeks, the majority of patients described a significant improvement of their quality of life. Work-up at 12 weeks revealed a significant reduction of serum biomarkers and LVOT gradients. In four patients, mavacamten needed to be temporarily paused due to clinical complaints or transient left ventricular ejection fraction deterioration below 50% with subsequent full recovery.

**Conclusion:**

We provide first insights into the usage of mavacamten in oHCM patients during the titration period in a real-world setting. Clinical findings are in line with previous clinical trials. In accordance with current recommendations, we highlight the need for standardized follow-up of patients on mavacamten treatment.

**Supplementary Information:**

The online version contains supplementary material available at 10.1007/s00392-024-02544-w.

## Introduction

Hypertrophic cardiomyopathy (HCM) is the most common primary cardiomyopathy and is characterized by a complex myocardial disorder caused by excessive actin-myosin cross bindings leading to hypercontractility, thickening of the left ventricular myocardium, diastolic dysfunction and elevated left ventricular filling pressure. Obstructive HCM (oHCM) is further characterized by an obstruction of the left ventricular outflow tract (LVOT) that occurs in up to 75% of HCM and may be unmasked only upon exercise test or Valsalva maneuver [1, 2]. So far pharmacological treatment was limited primarily to beta blockers or calcium channel blockers [1, 2], none addressing the underlying molecular disease mechanism. Besides medical options, oHCM with significant LVOT gradients can be treated with invasive septum reduction procedures such as surgical myectomy or transcoronary ablation of septal hypertrophy (TASH) [1, 2].

Lately, mavacamten, a first-in-class, cardiac-specific myosin inhibitor, emerged as a new therapy option for oHCM. Mavacamten diminishes the actin-myosin cross binding formation, thereby improves myocardial energetics and reduces contractility [3]. Clinical phase 2 and 3 trials demonstrated the reduction of LVOT obstruction, reduced circulating biomarkers and improved exercise capacity. In addition, typical HCM-related symptoms such as dyspnea, angina and edema improved; overall, mavacamten was well tolerated [4, 5]. Mavacamten was approved by the US Food and Drug Administration (FDA) and the European Medicines Agency (EMA) for the treatment of symptomatic oHCM patients (NYHA classes II and III) [6, 7]. Due to the direct intervention with the actin and myosin cross binding formation, regular reevaluation of the left ventricular ejection fraction (LVEF) is recommended every 4 weeks during the initial titration phase lasting 12 weeks. In addition, it is suggested to adapt the initial dosing depending on genetic variants in cytochrome CYP2C19 that affect metabolism [7, 8].

Despite promising results from clinical trials concerning safety and effectiveness of mavacamten real-world experience of this new treatment option is lacking. Here we give first insight into a cohort treated with mavacamten in order to evaluate manageability, effectiveness and safety in an outpatient, and real-world clinical setting.

## Material and methods

22 consecutive patients with oHCM presenting to our outpatient clinic between March 2023 and June 2024 eligible for therapy with mavacamten according to current treatment recommendations (age > 18 years, unexplained LV hypertrophy ≥15 or ≥13 mm in familial HCM, no storage diseases such as Fabry’s disease, clinical presentation with dyspnea NYHA class II-III, left ventricular ejection fraction ≥55%, resting or provoked peak LVOT pressure gradients of ≥30 mmHg) were included.

Of these 22 patients, seven patients with arterial hypertension were additionally on stable off-label medication with Angiotensin-converting-enzyme-inhibitors (ACE-i) or Angiotensin-II receptor blocker (ATII-b), which does not represent common practice according to the guidelines. Therefore, these seven patients were excluded from primary analysis. One patient stopped mavacamten at week 8. The remaining 14 patients had a follow-up of 12 weeks.

According to the manufacturer’s recommendations genotyping for cytochrome P450 (CYP2C19) was performed to determine the correct individual starting dosage of mavacamten and therapy with mavacamten was not started until CYP2C19 status was available: Patients defined as poor metabolizer were started on 2.5 mg mavacamten per day, whereas patients with intermediate to normal metabolizer genotype were treated with 5 mg mavacamten per day. Echocardiography, resting electrocardiography (ECG), and blood sampling were performed prior to treatment initiation and four, eight, and 12 weeks after start of the medication. Echocardiographic assessment of provoked peak LVOT gradient was done after Valsalva´s maneuver. NYHA class and Kansas City Cardiomyopathy-12 (KCCQ-12) Score were assessed during visits as a heart failure-specific quality of life measure. As described before, the KCCQ-12 measures and quantifies symptoms, physical and social limitations, and quality of life [9]. Differences of 5 points or greater were considered to be clinically significant.

Additionally, we defined “complete response” as a composite including a LVOT gradients <30 mmHg and NYHA class I.

### Statistical analysis

For statistical analyses, we used GraphPad Prism 10.2.1. A Mann–Whitney-*U*-Test was used for statistical testing. Data points were depicted as box and whiskers (whiskers = min–max). *p* values < 0.05 were considered as significant.

## Results

### Characterization of the patient population

The 15 patients included in our primary analysis were 53.4 ± 16.3 years old; 60% were male patients. Clinically, most patients presented with dyspnea and NYHA class III (73.3%). 40% of patients suffered from angina pectoris and 33.3% from vertigo on exertion; 26.7% had a history of syncope in the past. Mean resting LVOT gradient was 42.2 ± 23.9 mmHg; in six patients resting LVOT gradient was <30 mmHg with elevated LVOT gradients under provocation (83.2 ± 34.4 mmHg). Mean peak LVOT gradient was 100.7 ± 38.6 mmHg before treatment indicating significant LVOT obstruction in all patients. The mean HCM-Risk-Score was 3.9 ± 2.3. Genetic testing for CYP2C19 variants was performed in all patients: four patients (26.7%) were classified as intermediate metabolizers, one patient (6%) as poor metabolizer status, and the remaining patients as normal metabolizers (67.3%). Most common cardiovascular comorbidities were hypertension (20%) and hypercholesterolemia (40%). Three patients (20%) had an implantable cardioverter-defibrillator. Three patients (20%) were previously treated with TASH (mean 7.7 ± 5.6 months; median 6 [IQR 11] months prior to mavacamten initiation) and diagnosed with recurrent and significant resting (40.0 ± 30.4 mmHg) and peak LVOT gradients (96.7 ± 28.9 mmHg).

All 15 patients were taking accompanying medication before therapy initiation. 13/15 patients were on ß-blocker treatment at baseline and dosages were 51 ± 26.3% of the daily maximum dosage of the corresponding ß-blocker; dosages were not changed within the 12-week initiation period. One patient was on verapamil (see also baseline characteristics, Table 1). The baseline characteristics of the subgroup of patients (*n* = 7) on off-label ACE-i or ATII-b therapy are summarized in Supplement Table 1.

**Table 1:** Baseline characteristics of patients included in primary analysis

	*n* = 15
Age (years)	53.4 ± 16.3
Sex, male	9 (60%)
BMI (kg/m^2^)	28.6 ± 6.0
oHCM	15 (100%)
HCM-Risk-Score	3.9 ± 2.3
Implantable cardioverter-defibrillator	3 (20%)
Medical history	
Atrial fibrillation	4 (26.7%)
TASH	3 (20%)
Hypertension	3 (20%)
Diabetes mellitus	0 (0%)
Hypercholesterolaemia	6 (40%)
Non sustained VTs	3 (20%)
Sudden cardiac death of a family member	3 (20%)
Pulmonary disease	1 (6%)
Clinical presentation at baseline	
Angina pectoris	6 (40%)
Vertigo on exertion	5 (33.3%)
NYHA class II	4 (26.7%)
NYHA class III	11 (73.3%)
Syncope	4 (26.7%)
Laboratory value at baseline	
NT-proBNP, median [IQR] (pg/ml)	722.0 [791.0]
Concurrent oHCM related medication	
Beta-blocker	13 (87%)
Calcium channel blockers (verapamil-type)	1 (6%)
Echocardiographic parameters at baseline	
Left interventricular septum-diameter diastolic (mm)	23 ± 8.5
Left ventricular ejection fraction (%)	60.4 ± 5.9
LVOT gradient, rest (mmHg)	42.2 ± 23.9
LVOT gradient, peak (mmHg)	100.7 ± 38.6
LAVI (ml/m^2^)	69.6 ± 25.9
Cytochrome P-4502C19*2 status	
Intermediate metabolizer	4 (26.7%)
Poor metabolizer	1 (6%)
Genetic testing for HCM related variances	9 (60%)
No pathogenic or likely pathogenic variance	5(55.6%)
Pathogenic or likely pathogenic variances	4 (44.4%)^a^

Data are mean ± SD, *n* (%), or *n*/*N* (%), unless otherwise stated

*BMI* body mass index, *oHCM* obstructive hypertrophic cardiomyopathy, *TASH* transcoronary ablation of septal hypertrophy, *VTs* ventricular tachycardia, *NYHA class* New York Heart Association Classification, *NT-proBNP* N-terminal pro–B-type natriuretic peptide, *LVOT* left ventricular outflow tract, *LAVI* left atrial volume index

^a^Pathogenic or likely pathogenic variances in the myosin-binding protein C3 (MYBPC3) gene *n* = 1, the troponin I3 (TNNI3) gene *n* = 1 and the myosin heavy chain 7 (MYH7) gene *n* = 2

### Treatment with mavacamten improves symptoms, has an impact on echocardiographic and electrocardiographic parameters and reduces NT-proBNP levels

**Fig. 1:**
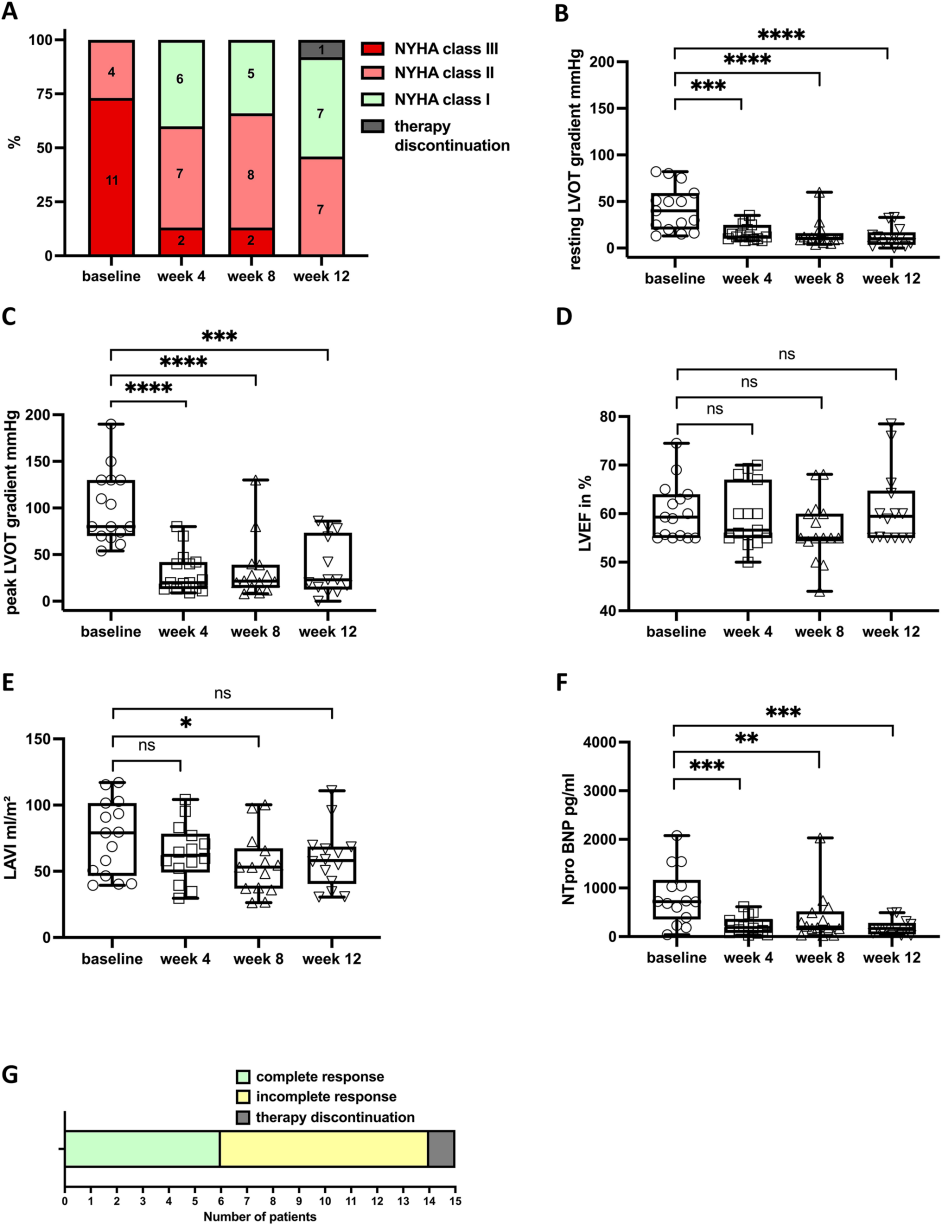
NYHA classes, echocardiographic parameters and serum biomarkers during the 12-week initiation period. NYHA classes at baseline and 4, 8 and 12 weeks after initiation of mavacamten (**A**) Resting LVOT gradients under mavacamten therapy at baseline and after 4, 8 and 12 weeks (**B**) peak LVOT gradients at baseline and under mavacamten therapy after 4, 8 and 12 weeks (**C**) LVEF in % at baseline and week 4, 8 and 12 after initiation of mavacamten (**D**) and LAVI ml/m^2^ at baseline and after 4, 8 and 12 weeks of therapy with mavacamten (**E**) NT-proBNP levels at baseline and after 4, 8 and 12 weeks of treatment with mavacamten. One outlier showed distinct higher NT-proBNP levels and was taken out of this graph. NT-proBNP level of this patient: baseline (9557 pg/ml), week 4 (6164 pg/ml), week 8 (4608 pg/ml), week 12 (2612 pg/ml) (**F**) Complete response was met by 6/14 patients (43%) after 12 weeks of therapy (**G**) Data from one patient who discontinued mavacamten therapy after 8 weeks was excluded from the 12-week analyses. Data is shown as box and whiskers (min to max). ns = not significant, * *p* ≤ 0.05, ** *p* ≤ 0.01, *** *p* ≤ 0.001, **** *p* ≤ 0.0001. NYHA class = New York Heart Association Classification. LVOT = left ventricular outflow tract. LVEF = left ventricular ejection fraction. LAVI = left atrial volume index. NT-proBNP = N-terminal pro–B-type natriuretic peptide

The majority of patients (10/15 patients; 67%) reported an improvement of at least one NYHA class after 4 and 8 weeks of treatment. Mavacamten therapy was discontinued in one patient after week 8; this patient was excluded from analysis at week 12. 86% of the remaining 14 patients reported consistent improvement of at least one NYHA class after 12 weeks of therapy. While 73.3% of all patients stated dyspnea according to a NYHA class III at baseline, 50% no longer suffered from dyspnea (NYHA class I) and 50% reported improved dyspnea (NYHA class II) at 12 weeks of treatment (Fig. 1A). The mean levels of KCCQ-12 were 57.6 ± 19.5 points at baseline and 68.3 ± 22.1 points after 8 and 70.6 ± 19 points after 12 weeks of therapy indicating an improvement of quality of life (mean change week 8: 3.9 ± 17.9 points; 95% CI [−7.2; 15.0], mean change week 12: 14.7 ± 12.1 points; 95% CI [7.8; 21.9]).

The mean resting and peak LVOT gradients decreased from 42.2 ± 23.9 to 100.7 ± 38.6 mmHg at baseline to 14.5 ± 13.9 and 33.4 ± 33.4 at 8 weeks and 12.2 ± 10.6 mmHg and 36.4 ± 30.8 mmHg at 12 weeks, respectively. Mean changes in resting and peak LVOT gradients were 27.7 ± 28.4 mmHg (95% CI [13.4; 42.1], *p* < 0.0001) and 69.6 ± 50.4 mmHg (95% CI [43.2; 96.0], *p* < 0.0001) at 8 weeks and 28.2 ± 28.9 mmHg (95% CI [12.4; 43.9], *p* < 0.0001) and 63.4 ± 59.4 mmHg (95% CI [31.1; 95.7], *p* = 0.0002) at 12 weeks, respectively (Fig. 1B, C). LVEF did not deteriorate over time (Fig. 1D), and we noticed a non-significant reduction of the left atrial volume index (LAVI) (Fig. 1E). In addition, the mean serum N-terminal pro–B-type natriuretic peptide (NT-proBNP) level significantly decreased from 1406.3 ± 2322.7 pg/ml (median: 722.0 pg/ml [IQR: 791.0]) at baseline to 672.8 ± 1197.9 pg/ml (median: 210.0 pg/ml [IQR: 383.0]) at 8 weeks and 358.5 ± 666.7 pg/ml (median 167.0 pg/ml [IQR: 223.0]) at 12 weeks. Mean changes in NT-proBNP were 733.4 ± 1362.6 pg/ml (95% CI [44.0; 1422.9], *p* = 0.019) at 8 weeks and 1104.9 ± 1751.1 pg/ml (95% CI [187.7; 2022.2], *p* = 0.0013) at 12 weeks (Fig. 1F). Complete response was met by 4/15 (27%) patients after 8 weeks and by 6/14 (43%) patients after 12 weeks (Fig. 1G). The subgroup of seven patients with off-label usage of ACE-i or ATII-b before and after mavacamten initiation depicted comparable responses to treatment (Supplement Fig. 1).

**Fig. 2:**
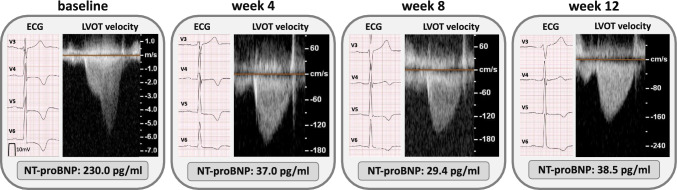
Exemplary presentation of T-wave inversion, LVOT velocity and NTproBNP levels in one representative patient with complete response during the 12-week titration period. Changes in T-wave inversions, LVOT velocity and NT-proBNP values at baseline, 4, 8 and 12 weeks under therapy with mavacamten. Peak LVOT gradients at baseline: 155 mmHg, at week 4: 12 mmHg, at week 8: 11 mmHg, at week 12: 12 mmHg. ECG calibration: paper speed 50 mm/s, calibration spike 10 mm/mV (indicated in baseline). LVOT velocity in m/s at baseline and cm/s at week 4, 8 and 12. ECG = electrocardiogram. LVOT = left ventricular outflow tract. NT-proBNP = N-terminal pro–B-type natriuretic peptide

Evaluation of resting ECGs revealed nine patients (60%) with inverted T-waves before therapy initiation. In seven out of nine patients, T-wave inversions were persisting to a lesser extent, while in two patients T-wave inversions were no longer detectable after 8 or 12 weeks of therapy. Figure 2 shows changes of T-wave inversions, reduction of LVOT velocity and serum NT-proBNP levels of one representative patient with complete therapy response during the 12-week initiation period.

### Reasons for dosage adaptation, pausing or discontinuation of mavacamten therapy during the 12-week initiation period

**Fig. 3:**
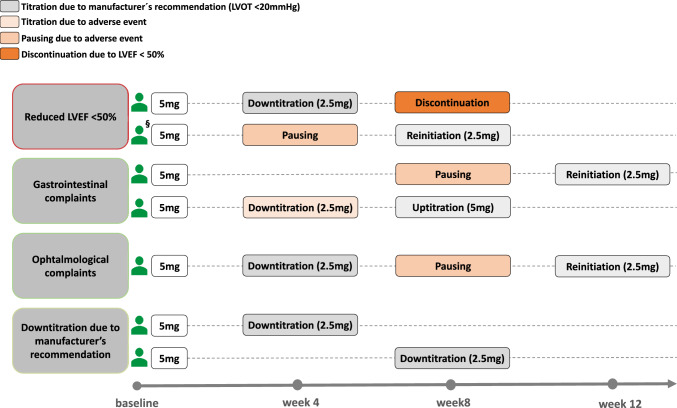
Reasons for changes in mavacamten dose and management over 12 weeks of therapy. ^§^ Indicating one patient with continued Angiotensin-Converting-Enzyme-Inhibition therapy

While therapy with mavacamten was generally well-tolerated in most patients, four patients (27%) paused treatment due to deteriorating LVEF (<50%, *n* = 1, week 8), gastrointestinal (*n* = 2 at week 4 and 8, respectively) and ophthalmological (*n* = 1, at week 8) complaints. Therapy was reinitiated at lower dosage in three of these four cases without any further side effects. The patient with persistent LVEF reduction was already on the lowest available mavacamten dose, thus therapy was discontinued after week 8. In two additional cases, the mavacamten dosage was reduced due to peak LVOT gradient reduction < 20 mmHg. In the subgroup of patients with continued off-label ACE-i or ATII-b therapy, treatment with mavacamten was paused in one additional case due to transient LVEF deterioration below 50% at week 4; therapy was recontinued at week 8 (Fig. 3).

## Discussion

With this analysis of 15 patients treated with mavacamten in an outpatient clinical setting, we provide insight into a real-world cohort describing safety and effectiveness during the initial titration period. Our data showed that mavacamten was generally well tolerated by this symptomatic oHCM cohort. A significant and lasting improvement of subjective clinical endpoints such as NYHA class and KCCQ Score were confirmed. According to previous findings of the EXPLORER-HCM and MAVA-LTE trials [4, 10], resting and peak LVOT gradients and serum levels of NT-proBNP were significantly and persistently reduced. In addition, we observed a trend of LAVI reduction after 12 weeks in the majority of patients, reflecting a potential reduction of left ventricular filling pressures in this early treatment phase.

The interference of mavacamten with the actin-myosin cross binding formation might potentially result in reduced LVEF. Therefore, all patients underwent echocardiographic reevaluation every 4 weeks—as recommended by the manufacturer [6]. Significant LVEF deterioration (LVEF < 50%) was documented in one case 8 weeks after therapy initiation. In this case, therapy was discontinued due to concerns about recurrent LVEF deterioration; the patient was already on the lowest mavacamten dose prior to the LVEF deterioration. In this case, echocardiographic reevaluation after week 12 further revealed a recurrent peak LVOT gradient of 75 mmHg and significant symptom burden (NYHA class III). The patient is now being evaluated for TASH.

In addition, mavacamten was temporarily discontinued in three patients due to elevated liver enzymes (in addition to temporary heavy alcohol abuse, *n* = 1), stomach pain (*n* = 1) and visual field loss (*n* = 1). The elevated liver enzymes normalized between week 4 and 8 under reduced mavacamten dosage (highest value at week 4: AST 3.4 and ALT 2.6 times elevated above laboratory threshold) and avoidance of alcohol; we were able to uptitrate mavacamten again afterwards. Stomach pain was most likely associated with a poorly controlled diabetes mellitus type II; symptoms disappeared after the intensified diabetes therapy. The remaining case reported marginal visual field loss with significant improvement after dose adaption.

The subgroup of patients with continued off-label use of ACE-i or ATII-b was carefully evaluated. The use of ACE-i or ATII-b could potentially increase LVOT gradients and does not represent common practice according to the European Society of Cardiology (ESC) guidelines [11–13]. Therefore, we highlight that mavacamten therapy in those cases was an off-label use. Accordingly, the cotreatment with antihypertensive medication has changed in our center and antihypertensive drugs independent from renin–angiotensin–aldosterone system are used for blood pressure control. In addition, ACE-i or ATII-b are discontinued and LVOT gradients are reevaluated after discontinuation and before initiation of mavacamten.

In summary, mavacamten is a safe and efficient therapy for patients with symptomatic oHCM. According to current recommendations, we monitored our cohort closely in order to adjust dosages, as well as for possible side effects. Therefore, we support the idea of a standardized follow-up including clinical evaluation, ECG, imaging, and serum biomarkers for all patients started on mavacamten. Due to the small sample size of our single center cohort, multicenter and multinational registers are urgently needed to further optimize therapeutic strategies and safety.

## Supplementary Information

Below is the link to the electronic supplementary material.

**Supplement Figure 1**: **NYHA classes, echocardiographic parameters and serum biomarkers during the 12-week initiation period in a subgroup of patients (n=7) with comedication of Angiotensin-converting-enzyme-inhibitor or Angiotensin-II receptor blocker. **NYHA classes at baseline and week 4, 8 and 12 after initiation of mavacamten **(A)** Resting LVOT gradients at baseline and under mavacamten therapy after 4, 8 and 12 weeks **(B)** Peak LVOT gradients at baseline and under mavacamten therapy after 4, 8 and 12 weeks **(C)** LVEF in % at baseline and week 4, 8 and 12 after initiation of mavacamten **(D) **LAVI ml/m² at baseline and after 4, 8 and 12 weeks of therapy with mavacamten **(E)** NT-proBNP levels at baseline and after 4, 8 and 12 weeks of treatment with mavacamten **(F) **Data is shown as box and whiskers (min to max). ns= not significant, * p ≤0.05, ** p≤ 0.01, *** p≤ 0.001, **** p≤ 0.0001 NYHA class=New York Heart Association Classification. LVOT=left ventricular outflow tract. LVEF=left ventricular ejection fraction. LAVI=left atrial volume index. NT-proBNP=N-terminal pro–B-type natriuretic peptide. (PDF 1378 KB)

**Supplement Table 1: Baseline characteristics of patients with Angiotensin-converting-enzyme-inhibitor or Angiotensin-II receptor blocker. **Data are mean±SD, n (%), or n/N (%), unless otherwise stated. ACE-i=Angiotensin-converting-enzyme-inhibitor, ATII-b=Angiotensin-II receptor blocker. BMI=body mass index. oHCM=obstructive hypertrophic cardiomyopathy. TASH=transcoronary ablation of septal hypertrophy. VTs=ventricular tachycardia. NYHA class=New York Heart Association Classification. NT-proBNP=N-terminal pro–B-type natriuretic peptide. LVOT=left ventricular outflow tract. LAVI=left atrial volume index. (DOCX 21 KB)

## Data Availability

The data that support the findings of this study are available on request from the corresponding author. The data are not publicly available due to their containing information that could compromise the privacy of research participants.
