# Supplementary figures and images for: Real-world experience in initiation of treatment with the selective cardiomyosin inhibitor mavacamten in an outpatient clinic cohort during the 12-week titration period
Source: Clin Res Cardiol. 2024 Oct 8;115(5):725–31. doi: 10.1007/s00392-024-02544-w (PMC13083493; doi:10.1007/s00392-024-02544-w)

Supplement Figure 1

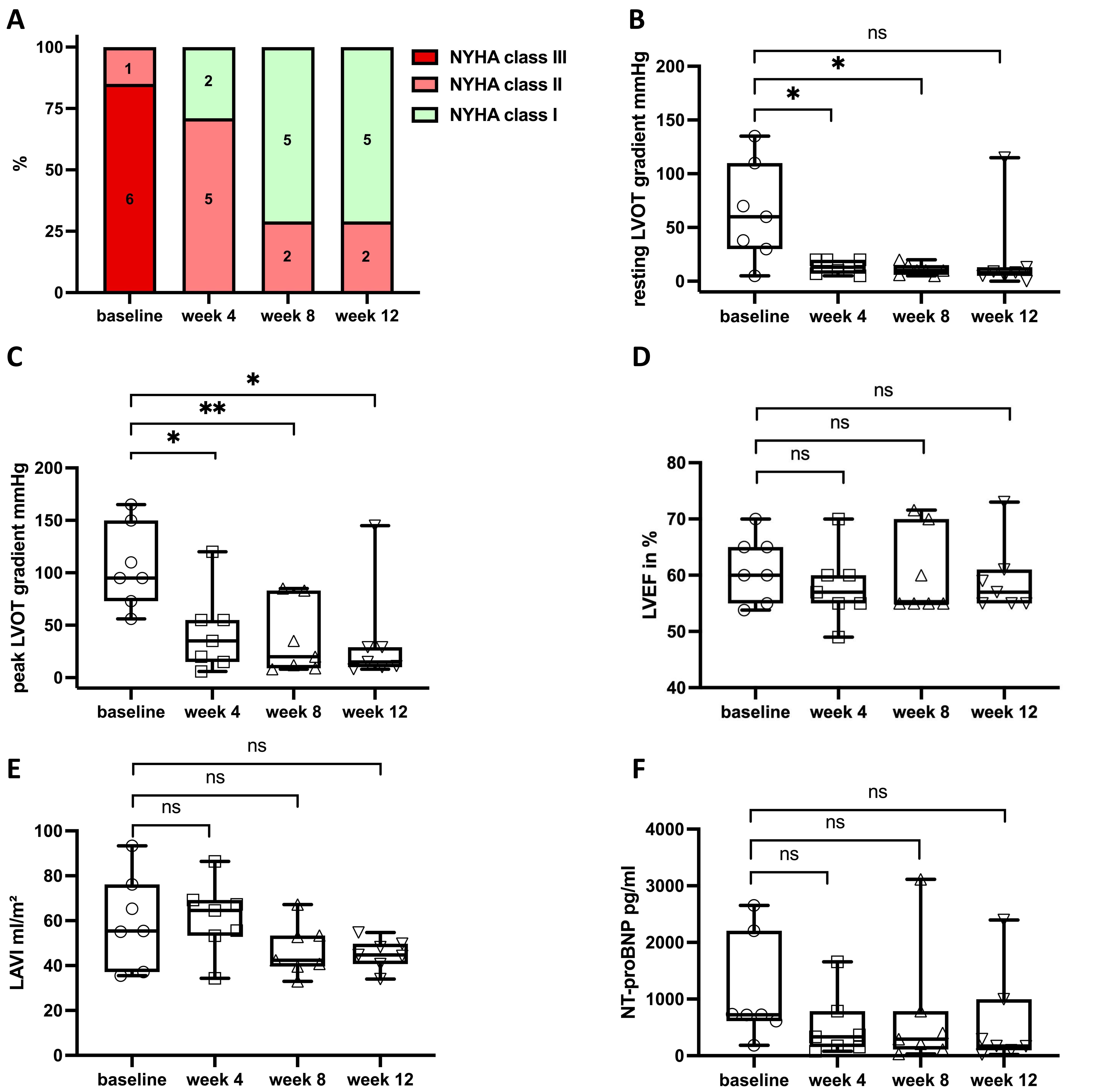

Supplement: Supplementary file 1 — Supplement Figure 1: NYHA classes, echocardiographic parameters and serum biomarkers during the 12-week initiation period in a subgroup of patients (n=7) with comedication of Angiotensin-converting-enzyme-inhibitor or Angiotensin-II receptor blocker. NYHA classes at baseline and week 4, 8 and 12 after initiation of mavacamten (A) Resting LVOT gradients at baseline and under mavacamten therapy after 4, 8 and 12 weeks (B) Peak LVOT gradients at baseline and under mavacamten therapy after 4, 8 and 12 weeks (C) LVEF in % at baseline and week 4, 8 and 12 after initiation of mavacamten (D) LAVI ml/m² at baseline and after 4, 8 and 12 weeks of therapy with mavacamten (E) NT-proBNP levels at baseline and after 4, 8 and 12 weeks of treatment with mavacamten (F) Data is shown as box and whiskers (min to max). ns= not significant, * p ≤0.05, ** p≤ 0.01, *** p≤ 0.001, **** p≤ 0.0001 NYHA class=New York Heart Association Classification. LVOT=left ventricular outflow tract. LVEF=left ventricular ejection fraction. LAVI=left atrial volume index. NT-proBNP=N-terminal pro–B-type natriuretic peptide. (PDF 1378 KB) [file 392_2024_2544_MOESM1_ESM.pdf]
