# Supplementary material for: Real-world experience in initiation of treatment with the selective cardiomyosin inhibitor mavacamten in an outpatient clinic cohort during the 12-week titration period
Source: Clin Res Cardiol. 2024 Oct 8;115(5):725–31. doi: 10.1007/s00392-024-02544-w (PMC13083493; doi:10.1007/s00392-024-02544-w)
Supplement: Supplementary file 2 — Supplement Table 1: Baseline characteristics of patients with Angiotensin-converting-enzyme-inhibitor or Angiotensin-II receptor blocker. Data are mean±SD, n (%), or n/N (%), unless otherwise stated. ACE-i=Angiotensin-converting-enzyme-inhibitor, ATII-b=Angiotensin-II receptor blocker. BMI=body mass index. oHCM=obstructive hypertrophic cardiomyopathy. TASH=transcoronary ablation of septal hypertrophy. VTs=ventricular tachycardia. NYHA class=New York Heart Association Classification. NT-proBNP=N-terminal pro–B-type natriuretic peptide. LVOT=left ventricular outflow tract. LAVI=left atrial volume index. (DOCX 21 KB) [file 392_2024_2544_MOESM2_ESM.docx]

**Supplement Table 1: Baseline characteristics of patients with Angiotensin-converting-enzyme-inhibitor or Angiotensin-II receptor blocker**

|  | n=7 |
| --- | --- |
| Age, years | 69.4 ± 9.9 |
| Sex, male | 3 (43%) |
| BMI kg/m² | 28.4 ± 3.2 |
| oHCM | 7 (100%) |
| HCM-Risk-Score | 2.4 ± 0.7 |
| Implantable cardioverter-defibrillator | 0 (0%) |
| Medical history |  |
| Atrial fibrillation | 1 (14.3%) |
| TASH | 4 (57.1%) |
| Hypertension | 7 (100%) |
| Diabetes mellitus | 0 (0%) |
| Hypercholesterolaemia | 6 (85.7%) |
| Non sustained VTs | 0 (0%) |
| Sudden cardiac death of a family member | 1 (14.3%) |
| Pulmonary disease | 2 (28.6%) |
| Clinical presentation at baseline |  |
| Angina pectoris | 2 (28.6%) |
| Vertigo on exertion | 1 (14.3%) |
| NYHA class II | 1 (14.3%) |
| NYHA class III | 6 (85.7%) |
| Syncope | 0 (0%) |
| Laboratory value at baseline |  |
| NT-proBNP, median [IQR], pg/ml | 720 [1208.5] |
| Concurrent medication |  |
| Beta-blocker | 7 (100%) |
| Calcium channel blockers (verapamil-type) | 1 (14.3%) |
| ACE-i/ATII-b | 7 (100%) |
| Echocardiographic parameters at baseline |  |
| Left interventricular septum-diameter diastolic, mm | 19.4 ± 2.4 |
| Left ventricular ejection fraction, % | 61 ± 6 |
| LVOT gradient, rest, mmHg | 64 ± 45.7 |
| LVOT gradient, peak, mmHg | 106.3 ± 39.3 |
| LAVI, ml/m² | 59.7 ± 20.7 |
| Cytochrome P-4502C19*2 status |  |
| intermediate metabolizer | 3 (42.9%) |
| poor metabolizer | 1 (14.3%) |
| Genetic testing for HCM related variances | 2 (28.6%) |
| No pathogenic or likely pathogenic variance | 2(100%) |
| Pathogenic or likely pathogenic variances | 0 (0%) |
